# Validation Study of QSAR/DNN Models Using the Competition Datasets

**DOI:** 10.1002/minf.201900154

**Published:** 2019-12-18

**Authors:** Yoshiki Kato, Shinji Hamada, Hitoshi Goto

**Affiliations:** ^1^ Department of Computer Science and Engineering Toyohashi University of Technology 1-1 Hibarigaoka, Tempaku cho Toyohashi, Aichi 441-8580 Japan

**Keywords:** Merck Molecular, Activity Challenge, Chainer Chemistry, Deep Neural Network, Machine Learning

## Abstract

Since the QSAR/DNN model showed predominant predictive performance over other conventional methods in the Kaggle QSAR competition, many artificial neural network (ANN) methods have been applied to drug and material discovery. Appearance of artificial intelligence (AI), which is combined various general purpose ANN platforms with large‐scale open access chemical databases, has attracting great interest and expectation in a wide range of molecular sciences. In this study, we investigate various DNN settings in order to reach a high‐level of predictive performance comparable to the champion team of the competition, even with a general purpose ANN platform, and introduce the Meister setting for constructing a good QSAR/DNNs model. Here, we have used the most commonly available DNN model and constructed many QSAR/DNN models trained with various DNN settings by using the 15 datasets employed in the competition. As a result, it was confirmed that we can constructed the QSAR/DNN model that shows the same level of R2 performance as the champion team. The difference from the DNN setting recommended by the champion team was to reduce the mini‐batch size. We have also explained that the R2 performance of each target depends on the molecular activity type, which is related to the complexity of biological mechanisms and chemical processes observed in molecular activity measurements.

## Graphical abstract

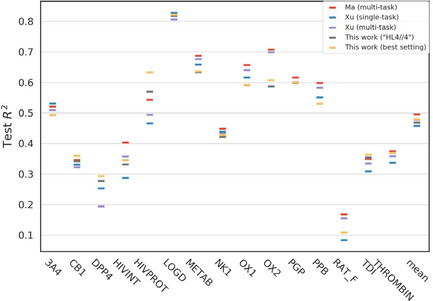

## 1 Introduction

The usefulness of deep leaning techniques in computer‐assisted drug discovery and design is highly expected for its potential to dramatically improve the prediction accuracy of the qualitative structure‐activity relationship (QSAR) methodology.[1] In fact, deep neural networks (DNN) approaches in the Kaggle “Merck Molecular Activity Challenge (MMAC)” competition held in 2012 [2] and the post analysis [3, 4] showed better predictive performance than the conventional methods like random forest (RF) and support vector machine (SVM). The success of the QSAR/DNN approaches was a significant impact on many researchers in chemistry and pharmaceutical fields, and created a major trend that followed to apply many recent artificial neural network (ANN) techniques to drug and material discovery, such as Recurrent Neural Network (RNN) with long short‐term memory cells (LSTM) and Convolutional Neural Network (CNN) [5–8].

Traditional QSAR, multiple regression methods using a relatively small candidate dataset for one target, has been evolving to contribute to the final refinement of more effective drug and material candidate compounds by introducing physicochemical properties, quantum chemical parameters, and 3D‐stracture descriptors, with strong support of ligand docking approaches and theoretical molecular simulations [9–12]. On the other hand, the QSAR/DNN, which must rely on using large and diverse dataset, is expected as the latest tools to be able to contribute to the initial mining of exploring the lead candidates, even for non‐experts of drug and material discovery. Especially as a recent movement, database sites containing a huge amount of molecular data and activity‐property information, such as ChEMBL [13, 14] and PubChem,[15] have appeared on the Internet. In addition, there are openly available general‐purpose artificial neural network (ANN) platforms and the related libraries, such as TensorFlow, [16] Microsoft Cognitive Toolkit (CNTK), [17] Therano, [18] Chainer [19], Keras [20]. In this situation, even non‐experts of informatics or molecular science might expect to generate QSAR/DNN models with reasonable predictive performance by using them. In fact, our first QSAR/DNN attempt, which was used a general purpose ANN platform with the same datasets employed in the QSAR competition, was able to achieve enough predictive performance closed to that of the competition champion team [21].

Our attempt [21] prompted us some questions. Particularly, the following two:

1. There may be other DNN settings that can dramatically improve predictive performance.
2. It remains unclear the reasons why only one target achieved significant good predictive performance for test set, but many others showed only moderate performance and some were poor predictions.

After our report on the attempt, [21] we have examined further challenges to improve the predictive performance of the QSAR/DNN model. In this paper, we focus on the two questions mentioned above and analyze some of the results of our ongoing studies. Especially, we report here our investigations to find the Meister setting that can achieve a good predictive performance comparable to that of the champion team in the competition even when a common practice DNN model implemented in a general purpose ANN platform is used. Here, according to the protocol in the competition, each of the QSAR/DNN models for 15 targets is optimized by using the training set of each dataset, and its predicted performance is evaluated based on the R2 value of the test set. In addition, the DNN settings that give the best predictive performance for each target are also investigated, and the relationship between the limit of predictive performance and the ability of molecular descriptors are discussed in terms of the complexity of molecular activity measurements.

## 2 Methods

### 2.1 Data Sets

In this study, the datasets of 15 targets provided as the Supporting Information (SI) for the paper on the post‐analysis of the MMAC QSAR competition [3] are used. Each target dataset is divided into training and test sets along observed time series [22], and each set is given as a matrix composed of a fingerprint vector (molecular descriptors) and an activity value for each molecule. Table 1 shows the number of molecules in training and test sets of each target dataset, and the number of elements of each molecular fingerprint vector (size of the vector). We have confirmed that the numbers of molecules in training and test sets are exactly identical with the SI (but does not match to values in Table 1 of the post‐analysis paper) [3]. The only alignment we did is to unify the number of elements of the molecular fingerprint vectors between training and test sets for each target. By unifying the size of fingerprint vectors of each target, we can quickly evaluate the R2 performance of the test set for each epoch in iterations of QSAR/DNN optimization using the training set.

**Table 1:** Summary of Datasets.

Target	Description and unit of activity value	number of molecules^c^		number of descriptors merged
		Training set	Test set	
3A4	CYP P450 3A4 inhibition, pIC_50_^a^	37,241	12,338	9,491
CB1	binding to cannabinoid receptor 1, pIC_50_^a^	8,716	2,907	5,877
DPP4	inhibition of dipeptidyl peptidase 4, pIC_50_^a^	6,148	2,045	5,203
HIVINT	inhibition of HIV integrase in a cell based assay, pIC_50_^a^	1,815	598	4,306
HIVPROT	inhibition of HIV protease, pIC_50_^a^	3,212	1,072	6,274
LOGD	logD measured by HPLC method	37,388	12,406	8,921
METAB	percent remaining after 30 min microsomal incubation	1,569	523	4,505
NK1	inhibition of neurokinin1 (substance P) receptor binding, pIC_50_^a^	9,965	3,335	5,803
OX1	inhibition of orexin 1 receptor, p*K*i^b^	5,351	1769	4,730
OX2	inhibition of orexin 2 receptor, p*K*i^b^	11,151	3704	5,790
PGP	transport by *p*‐glycoprotein, log(BA/AB)	6,399	2093	5,135
PPB	human plasma protein binding, log(bound/unbound)	8,651	2,899	5,470
RAT_F	log(rat bioavailability) at 2 mg/kg	6,105	1,707	5,698
TDI	time dependent 3A4 inhibitions, log(IC50 without NADPH/IC50 with NADPH)	4,165	1382	5,945
THROMBIN	human thrombin inhibition, pIC_50_^a^	5,059	1,698	5,552

^a^ pIC_50_=−log(IC_50_) M ^b^ p*K*i=−log(*K*i) M ^c^ The number of molecules are included in the supporting information of the post‐analysis paper [3].

### 2.2 Molecular Descriptors

A molecule in a dataset is described as a vector, called molecular fingerprint (MFP), whose elements are natural numbers including zero, composed of counts of the unique substructures of a molecule. MFP, which was employed in the QSAR competition, is generated by simply unifying two vectors made from two kinds of molecular descriptors, Atom Pair (AP) [23] and Binding Property Pair (BP) [17]. Both descriptors first assign atom types to all atoms except for hydrogen, and classify into the one‐dimensional substructures according to the combination of the atom types for two atoms and the number of bonds connecting those atoms via minimal path:

$$\left(Atom\;type\;i\right)-\left(Distance\;in\;bonds<n\right)-\left(Atom\;type\;j\right)$$

where the atom type of AP method is classified by the element, valence bonding number except for hydrogen, and number of π electrons. BP method is also called a donor‐acceptor pair (DP) approach [24], and BP atom type is classified into seven categories: cation, anion, neutral donor, neutral acceptor, polar, hydrophobic, and other.

Each element of the descriptors is the number of appearances of each one‐dimensional substructure in a molecule, or zero if not. However, in the 15 datasets provided by the SI of the post‐analysis paper, [3] some zero elements are omitted in advance, because the corresponding substructure does not appear in any molecule in each training set and test set. For this reason, the size of fingerprint vector differs between the training set and the test set in each target's dataset. In this paper, by adding zero elements corresponding to the missing substructures, the size of fingerprint vector in the training set and the test set in each target is aligned to the same size, as mentioned above.

### 2.3 Molecular Activity

Activity data provided from the SI of the post‐analysis paper [3] should be noted. Activity data of 15 targets can be roughly classified into six or seven types, which are pIC50, pKi, log D, remaining percentage, logarithmic value of ratio between comparable biochemical processes (transporting both directions and receptor bound/unbound), and logarithmic value of bioavailability, as shown in Table 1. Ranges of these values are, roughly saying, within 4 to 10 for pIC50 and pKi, 0 to 5 for log D, 0 to 3 and −0.5 to 1.5 for PPB and PGP, respectively, and 0 to 2 for bioavailability. And the remaining incubation rate (%) in METAB is naturally in the range of 0–100. Because these range differences may affect the results in machine learning, in a practical sense, they are sometimes normalized in advance. However, these activity values themselves are numerical values that were already subject to logarithmic transformation in the derivation process, so further normalization of activity values are not performed in this study.

It should also be noted that these activity values may not be quantitatively correct due to the limited concentration range considered in the activity measurement. For example, according to explanation by the providers of the activity data, [3, 22] all concentrations above a certain level are given as constants (e. g. IC50=30 μM or ‐log (IC50)=4.5). This seems to usually have undesirable influence on machine learning, because DNN model may misunderstand that different molecules (different fingerprint vectors) have the same activity value. However, according to a post‐analysis of the champion team, the R2 performance for test sets tends to improve even if including a lot of molecules having the same activity value [3]. We set this aside to focus on constructing the QSAR/DNN models in our environment with reference to the champion team recommendations, and not discuss it in this paper.

### 2.4 Metrics

In this study, the QSAR/DNN model is optimized using the training set of a target dataset, and its prediction performance is evaluated by the squared Pearson correlation coefficient R2 between observed and predicted activities for the test set:

(1) $$R^{2}=\frac{{\left[\sum_{i=1}^{N}\left(x_{i}-\bar{x}\right)\left(y_{i}-\bar{y}\right)\right]}^{2}}{\sum_{i=1}^{N}{\left(x_{i}-\bar{x}\right)}^{2}\sum_{i=1}^{N}{\left(y_{i}-\bar{y}\right)}^{2}}$$

where xi is the observed activity value of molecule i, yi is the corresponding predicted activity value, and N is the number of molecules in the test set. For each target, the optimization of QSAR/DNN model is performed five times using each DNN setting, and the average of five R2 values is used as the metric for the predictive performance of the QSAR/DNN model. This metric of “R2 performance“ is the same as that of two post‐analysis papers by Ma et al. [3] and Xu et al. [4], and was also employed in the Kaggle QSAR competition.

On the other hand, MSE is the mean square error of observed and predicted activity values (Eq. (2)) and adopted to optimize QSAR/DNN model by using training set:

(2) $$MSE=\frac{\sum_{i=1}^{N}{\left(x_{i}-y_{i}\right)}^{2}}{N}$$

where N is the number of molecules in the training set.

MSE is not suitable for comparison among datasets, but can represent the quantitative correlation between observed and predicted activity values. Therefore, in this study, QSAR/DNN model optimized for the training set is evaluated exclusively by the R2 performance of the test set, while MSE for the training set is used as the target value (loss function) in optimization. Evaluation using both R2 and MSE or RMSE is very important, but it needs more detailed analyzes and leads to more complicated discussion, so, we discuss QSAR/DNN models based on R2 performance in this paper.

### 2.5 DNN sSttings for QSAR/DDN Model

**Figure 1:**
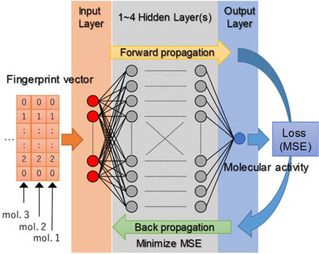
Illustration of a single‐task optimization of a fully connected feedforward network with one or more hidden layers.

In this study, we perform a single‐task optimization of a fully connected feedforward network with one or more hidden layers, which is implemented in a publicly available ANN framework, “Chainer”, that we have employed here (Figure 1). In order to apply this general purpose ANN to QSAR model optimization, it is necessary to investigate extensively the user controllable parameters of DNN settings that is typically called hyperparameters. Fortunately, the MMAC Champion Team has suggested their recommended DNN setting, mainly based on the results of the multitask DNN model [3, 4]. To make our suitable single‐task QSAR/DNN model, we examine various DNN settings with reference to the recommendation by the champion team. Table 2 shows the items of DNN settings examined in this study: the number of hidden layers, mini‐batch size, and logarithmic transformation of input data (molecular descriptors). Other items different from the champion team‘s recommendation are in that the number of epochs has increased up to 1000 cycles and Adam [25] has been adopted as a backpropagation learning (optimization) algorithm. All settings were tried at least 5 times for all target datasets.

**Table 2:** List of DNN settings.^*a*^

DNN setting	Number of Hidden Layers	Number of Neurons in each Layer	Dropout (%) in each Layer	Minibatch size	Log Transform
HL4	4	4000, 2000, 1000, 1000	25, 25, 25, 10	100	no
HL3	3	4000, 2500, 1500	25, 25, 10	100	no
HL2	2	6000, 2000	25, 10	100	no
HL1	1	8000	10	100	no
HL1/2	1	4000	10	100	no
HL1/4	1	2000	10	100	no
HL1/8	1	1000	10	100	no
HL1/16	1	500	10	100	no
HL4//2	4	4000, 2000, 1000, 1000	25, 25, 25, 10	50	no
HL4//4	4	4000, 2000, 1000, 1000	25, 25, 25, 10	25	no
LOG HL4	4	4000, 2000, 1000, 1000	25, 25, 25, 10	100	yes
LOG HL4//2	4	4000, 2000, 1000, 1000	25, 25, 25, 10	50	yes
LOG HL4//4	4	4000, 2000, 1000, 1000	25, 25, 25, 10	25	yes

^*a*^ HL*n* differs in the number of hidden layers, where n is the number of hidden layers. HL1/*m* means that the number of nodes in single hidden layer is equal to (1/*m*)x8000. HL4//*k* indicates that the mini‐batch size is changed to 100/k.

The standard DNN setting in this study is “HL4” as follows:

- DNN model should have four hidden layers, each with 4000, 2000, 1000, and 1000 neurons.
- The dropout rate is 0 % for the input layer and 25, 25, 25, and 10 % for the hidden layer, respectively.
- The activation function is the rectified liner unit (ReLU).
- DNN model must be initialized with random numbers without unsupervised pre‐training.

The range of DNN settings we investigate in this study is as follows (see also Table 2):

- “HL4”, “HL3”, “HL2”, “HL1”: the total number of neurons is keep to 8000, and the number of the hidden layers is changed.
- “HL1”, “HL1/2”, “HL1/4”, “HL1/8”: the number of the neurons in simple NN with the single hidden layer is changed.
- “HL4”, “HL4//2”, “HL4//4”: in training DNN with the four hidden layer, the mini‐batch size is changed.
- “LOG HL4”, “LOG HL4//2”, “LOG HL4//4”: the mini‐batch size for DNN training with the input logarithm transformation is changed.

The setting closest to the recommendation of the champion team is “LOG HL4”. In order to confirm the effect of the original descriptor, we have tried many settings without logarithmic transformation, here.

**Figure 2:**
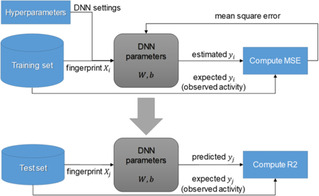
Workflow of optimization and evaluation for our QSAR/DNN model.

In this study, the R2 value of the test set can be quickly calculated for each training repetition, “epoch”, because of aligning the size of fingerprint vectors for the training and test set of each dataset. We therefore optimize our QSAR/DNN model using a DNN setting up to 1000 epochs, calculate the R2 value of the test set based on the instant DNN model of each epoch, and after optimization, record the best R2 value in 1000 epochs. The DNN optimization is repeated five times by using randomly initialized DNN parameters, and then, the average of their highest R2 values is evaluated as the R2 performance of the DNN setting. Although test set evaluation is usually not applicable to blind tests or practical applications, we use the R2 performance for evaluation of DNN settings in order to avoid the overfitting problem that require more complicated discussions.

## 3 Results

### 3.1 The best *R* ^2^ Performances

**Figure 3:**
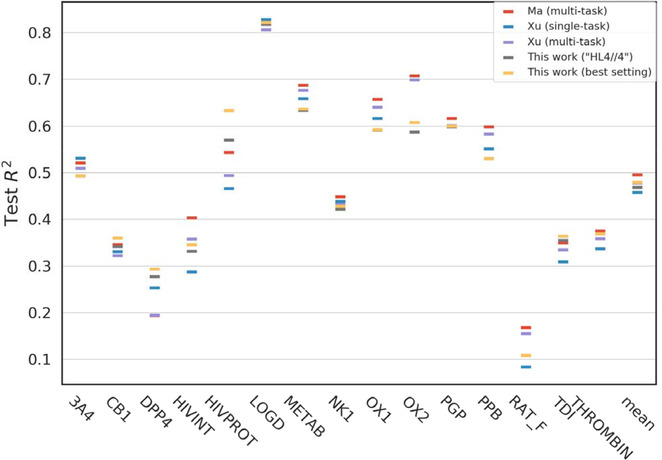
Comparison of *R* ^*2*^ performances of QSAR/DNN models. There are five entries comparing the median *R* ^*2*^ values from the multi‐task DNNs of Ma et al. [3], the single‐ and the multi‐tasks of Xu et al. [4], the average *R* ^*2*^ values from the single‐task of the best setting on overall average, the best *R* ^*2*^ collections of this work for each of 15 targets datasets, and also their averages.

Figure 3 shows a comparison of R2 performances of five QSAR/DNN models from multi‐task of Ma et al., [3] single‐ and multi‐tasks of Xu et al. [4], and two single‐tasks of us. In this figure, one of our results is the R2 performances of the QSAR/DNN models using the “HL4//4” setting, which showed the best value, 0.469, in the average R2 performance for overall 15 targets. Another one is a collection of the best R2 performance of each target when using various DNN settings, and the overall mean, 0.479, is obviously better than that of the “HL4//4” setting. In terms of the predictive performance of single‐task QSAR/DNN models, our result was better than that of Xu et al. [4] and was also close to that of multi‐tasks models of Ma et al. [3]. It should be noted that these performances were results of adopting different platforms and optimization algorithms, and additionally, our results have been shown as the average of 5 runs as mentioned above, while their results were presented as the median of more 20 runs. Therefore, what is important here is not that our results were better than those of the champion teams or worse, but that we were able to reproduce the trend of R2 performances similar to theirs, as well as our previous attempt. [21] In other words, we confirmed that both QSAR/DNN models optimized by using different ANN platforms and optimization methods were essentially equivalent.

### 3.2 R2 Performances for Each DNN Settings

We explain our results in a little more detail. Table 3 shows R2 performances of 15 targets when using various DNN settings. The overall average R2 performance of all targets for each setting and the standard deviation of R2 performances of each target for all settings are shown in the last column and row, respectively.

**Table 3:** *R* ^2^ performances of QSAR/DN models when using various DNN settings.^*a*^

Parameter Setting	3A4	CB1	DPP4	HIVINT	HIV PROT	LOGD	METAB	NK1	OX1	OX2	PGP	PPB	RAT_F	TDI	THROM BIN	Average^*b*^
HL4	0.4877	0.3486	0.2677	0.2778	0.5589	0.8195	0.6362	0.4229	0.5898	0.5796	0.5959	0.5218	0.1041	0.3604	0.3643	0.4624
HL3	0.4715	0.3529	0.2547	0.2784	0.5592	0.8215	0.6344	0.4193	0.5807	0.5709	0.5935	0.5153	0.0929	0.3626	0.3660	0.4583
HL2	0.4654	0.3417	0.2538	0.2970	0.5674	0.8189	0.6327	0.3965	0.5787	0.5800	0.6000	0.5143	0.0979	0.3641	0.3648	0.4582
HL1	0.4569	0.3481	0.2552	0.2523	0.5557	0.8196	0.6232	0.4044	0.5755	0.5693	0.5773	0.4978	0.0867	0.3027	0.3366	0.4441
HL1/2	0.4638	0.3408	0.2467	0.2446	0.5500	0.8202	0.6227	0.4064	0.5732	0.5700	0.5752	0.5006	0.0869	0.3192	0.3441	0.4443
HL1/4	0.4535	0.3473	0.2479	0.2318	0.5546	0.8179	0.6226	0.4093	0.5772	0.5631	0.5756	0.5017	0.0893	0.3295	0.3370	0.4439
HL1/8	0.4626	0.3352	0.2448	0.2208	0.5575	0.8160	0.6210	0.4025	0.5735	0.5727	0.5709	0.5020	0.0940	0.3300	0.3496	0.4435
HL1/16	0.4556	0.3498	0.2498	0.2299	0.5550	0.8158	0.6200	0.4053	0.5753	0.5643	0.5662	0.4935	0.0738	0.3263	0.3464	0.4418
HL4//2	0.4912	0.3598	0.2666	0.2997	0.5475	0.8181	0.6330	0.4206	0.5921	0.5989	0.5923	0.5298	0.1022	0.3570	0.3686	0.4652
**HL4//4**	**0.4930**	**0.3412**	**0.2771**	**0.3317**	**0.5694**	**0.8183**	**0.6341**	**0.4218**	**0.5916**	**0.5877**	**0.5984**	**0.5304**	**0.1088**	**0.3549**	**0.3690**	**0.4685**
LOG HL4	0.4830	0.3453	0.2720	0.3175	0.6333	0.8111	0.6194	0.4130	0.5816	0.6008	0.5896	0.5136	0.0955	0.3560	0.3211	0.4635
LOG HL4//2	0.4827	0.3392	0.2929	0.3231	0.6074	0.8107	0.6255	0.4193	0.5869	0.6027	0.5863	0.5236	0.0877	0.3549	0.3336	0.4651
LOG HL4//4	0.4875	0.3448	0.2934	0.3455	0.5963	0.8101	0.6285	0.4278	0.5800	0.6073	0.5881	0.5259	0.1001	0.3511	0.3384	0.4683
Std. Dev.^*c*^	0.0145	0.0064	0.0167	0.0421	0.0260	0.0038	0.0062	0.0096	0.0068	0.0156	0.0111	0.0128	0.0092	0.0197	0.0158	0.0107

^*a*^ Mean *R* ^*2*^ value is calculated as the average for the maximum *R* ^*2*^ values of five optimizations using each target's training set. Number underlined is the best mean *R* ^*2*^ value for each target when using various DNN parameter settings. Numbers in bold are the mean *R* ^*2*^ values and the overall average when using the “HL4//4” setting that showed the best average of the mean R2 values for overall targets. ^*b*^ Overall average of the mean *R* ^2^ values for 15 targets. ^*c*^ Standard deviation of the mean *R* ^2^ values for all DNN parameter settings.

From the average R2 performances, it is clear that the DNN settings including four hidden layers, that are “HL4”, “HL4//2”, “HL4//4”, “LOG HL4”, “LOG HL4//2”, “LOG HL4//4”, were totally effective. The best performance setting was “HL4//4” as mentioned above, and its overall average R2 performance reached 0.469. “LOG HL4” that is closest to the setting recommended by the champion team, was the average R2 of 0.465, which was almost equivalent to the “HL4//4” setting.

The most important difference between these settings is from the mini‐batch size, that is used to divided into small groups of molecules in the training set. One training process for updating the QSAR/DNN model by backpropagation optimization (i. e. using Adam algorithm in this study) using all molecules in each mini‐batch is once completed when finishing about all mini‐batches. This is called “epoch”. Therefore, the number of DNN updates per epoch is inversely proportional to the minibatch size:

(NumberofDNNupdatesperepoch)=(Numberofmoleculesintrainingset)/(Mini-batchsize)

Our results suggest that reducing the mini‐batch size improves R2 performances of QSAR/DNN models, at least, in our ANN environment. This trend was also observed in the DNN settings with logarithmic transformations of input molecular descriptors, such as “LOG HL4”, “LOG HL4//2” and “LOG HL4//4”. However, any improvement effect by usage of the logarithmic transformation itself was not observed in those average R2 performances.

It may also be important to notice that when using some typical neural network settings with single hidden layer, the overall average R2 performances were relatively poor. This can be usually understood as a natural behaviour of artificial neural networks, or as to expect for deep learning. However, with regard to the target LOGD, there was almost no decrease in R2 performances due to single hidden layer. This is explained in the next subsection.

### 3.3 R2 Performances of Each Target

The R2 performances of each target when using various DNN settings is investigated here. The most obvious observation from Figure 1 and Table 3 is that R2 performances of LOGD always showed the highest values >0.81 for overall targets, even with any DNN setting. Interestingly, the standard deviation of R2 performances of LOGD is less than 0.004, which means their performances are almost insensitive to DNN settings. More surprisingly, the DNN settings of “HL1” series, corresponding to a typical single hidden layered NN model showed better R2 performances than the logarithmic pre‐processed DNN settings of “LOG HL4” series. For example, even “HL1/16” with only 500 neurons in the single hidden layer showed a better R2 of 0.816, and that of “LOG HL4” was 0.811. The similar trend is also observed in METAB, which showed the second best R2 performance of all targets. In fact, the standard deviation of R2 performances of METAB was small, less than 0.01. The R2 performances of “HL1/16” and “LOG HL4” settings were 0.620 and 0.619, respectively., and there was almost no difference. From these observations, at least for LOGD and possibly also for METAB, it can be seen that the QSAR/DNN model may necessarily require neither multiple hidden layers nor a lot of neurons in a hidden layer to reproduce the output activity from the input molecular descriptors.

On the other hand, RAT_F showed the worst R2 performance for all targets. In particular, the reduction of the number of hidden layers and the number of neurons decreased the R2 performances. However, even if their numbers were increased, no significant improvement could be expected, and the best R2 performance was only 0.109 of “HL4//4”. Using any DNN setting we investigated in this work, it was impossible to predict the activity of RAT_F.

We can observe interesting trends in R2 performance for the other targets as well. For example, targets that predict the activity of pIC50 or pKi, which are closely related to each other, showed R2 performances of around 0.5. Looking more closely, the targets OX1 and OX2 with the activity of pKi were over 0.5 in R2 performance, while the targets with pIC50, except for HIVPROT, were less than 0.5 using any DNN setting. In the cases of PGP and PPB, the R2 performances showed 0.5 or higher. Here it can be found that the former required at least 5000 neurons and the latter needed at least four hidden layers.

From these observations, we noticed that the good R2 performance of the QSAR/DNN model in each target related to the type of the activity. At first, we thought that this dependency might be caused by the value range of the activity. However, we found that this was not the reason because both LOGD within the range of 0–5 and METAB of 0–100 showed higher R2 performance. Further analysis reveals that this problem is related to the complexity of the biological mechanisms and chemical processes involved in activity measurements. In the next section, we are going to consider R2 performance due to differences in activity types.

## 4 Discussion

What does it mean that R2 performance related to the molecular activity type?

QSAR/DNN models constructed in this study can generate predicted molecular activity values as higher‐order features from molecular fingerprints composed of molecular descriptors as primitive features. In other words, the QSAR/DNN model for each activity type is thought to implicitly include intermediate features related to the biological and/or chemical phenomenon leading to molecular activity. Unfortunately, for now, these intermediate features cannot be presented in human understandable format. If the R2 performance does not improve any more in the range of DNN settings investigated here, two factors must be considered for the question. If R2 performance does not improve any more in the range set in this study, there are two possible causes. One is that the output molecule activity is too complex for the input molecule fingerprint, and the other is that the input molecule fingerprint is too simple for the output molecular activity, so it may be not possible to construct some appropriate intermediate features. In this section, we will consider the relationship between R2 performance and molecular activity type in these two perspectives.

### 4.1 Limit of QSAR/DNN Models in this Work

Molecular activity types are generally designed so that only the biological or chemical effect of the molecule can be evaluated by determining the ratio of two biological mechanisms or chemical processes that are related but different or in equilibrium. For example, the active types of LOGD and RAT_F, which showed the best and the worst R2 performance, respectively, are going to be compared here. The activity type of LOGD is the log D, that is typically determined as an n‐octanol/water distribution coefficient and represents the hydrophobicity of the ionizable molecule. [26,27] On the other hand, the activity type of RAT_F is the bioavailability based on in vivo experiments, that is the ratio of the AUC (Area Under the blood concentration‐time curve) when the molecule is administered non‐intravenously and intravenously. However, log D is one of the molecular properties in terms of chemical processes in solubility for n‐octanol and water, whereas bioavailability evaluates the pharmacokinetics of non‐intravenously administered molecules, where many biological mechanisms in vivo are involved. That is, the former is a difference between simple chemical processes, and the latter is an activity measurement via the most complex phenomena in all active types in this study. Therefore, it is reasonable to think that the complexity (or simplicity) strongly affected on the differences in R2 performance. In order to improve R2 performance of RAT_F, it may be necessary to use more complex DNN model than our models adopted in this paper.

Furthermore, we are going to focus on the molecular activity types, pIC50 and pKi, that showed intermediate range in R2 performance. IC50 and Ki are measured by in vitro experiments, and both are evaluated on the premise of a competitive inhibition mechanism between the substrate and the inhibitor ligand molecule at the active site of the target enzyme reaction. IC50 is the concentration of molecule producing 50 % inhibition for the activity of the substrate against the target enzyme, and that depends on the concentration of the target enzyme, inhibitor molecule, and the substrate molecule along with other experimental conditions. On the other hand, Ki is the dissociation constant of the enzyme‐inhibitor complex, and is usually the quantitative feature of the inhibitor molecule called the inhibition constant. These two types of activity can be related by the following Cheng‐Prusoff equation, that is, in the case of a single‐substrate enzyme reaction that follows a simple biological mechanism [28,29]:

$${IC}_{50}=K_{i}\left(1+\left[S\right]/K_{m}\right)$$

where [S] is the concentration of the substrate molecule and Km is the Michaelis constant of the substrate, that is corresponding to, the substrate concentration at 1/2 maximum enzyme reaction rate. From this equation, we can understand that IC50 depends on the concentration of the substrate molecule. [30] In this study, the experimental conditions and measurement environment for the activity values of the dataset are unknown, so it cannot be stated clearly. However, if IC50 values in the dataset include such enzyme and substrate concentration dependency, it is understandable that R2 performance of pIC50 type may be inferior to that of Ki. Therefore, the predictive performances of 3 A4 and HIVPROT with pIC50 type were relatively better (R2≥0.5), which may had included a large number of the activity values measured under established experimental conditions.

Detailed descriptions of the other activity types are omitted here. However, at least from the analysis of the activity types of log D, bioavailability, pIC50 and pKi, it can be understood that their R2 performances depend on the biological mechanisms or chemical processes involved in the activity measurements. In fact, the activity type of the simple relative solubility showed the highest R2 performance in the simplest NN model with a single hidden layer. The R2 performances within the middle range were shown in the activity type of enzyme reaction constants measured in vitro, and the worst performance was the bioavailability type by in vivo experiment. In other words, the R2 performance peaks in the latter two active types may suggest that the QSAR/DNN models attempted in this study have reached a certain limit.

One way to break the limit and to improve the performance would be to deepen the DNN model by increasing the number of hidden layers and neurons in proportion to the complexity of the activity type. However, to perform this improvement practically, it is necessary to increase the number of activity data and computing power. Therefore, if the amount of data that can be used for machine learning is not sufficient, the depth and complexity of the network that can be employed are limited. Nonetheless, the performance improvement seems to be very small in our experience.

### 4.2 Limit of Descriptors

One‐dimensional substructure descriptors adopted in the competition and also in this study were able to reproduce property based on relatively simple chemical processes such as log D (R2>0.8), but not enough to represent molecular activities of in vitro type involving some complex biological mechanisms (R2∼0.5). Although one reason is that, as mentioned above, the QSAR/DNN model has reached its limit, the other limit of what can be represented by the descriptors should have also influenced the predictive performance. It is reasonable to think that the one‐dimensional substructure descriptors only provides structural information of the ligand molecule to the QSAR/DNN model, and that is insufficient contribution to predict the activity values measured as a result of complex mechanisms involving the target enzyme and its substrate.

In order to overcome the limit of the substructure descriptors and then improve prediction performance, it is effective to exchange the one‐dimensional descriptors to higher‐order features. For example, log D, which is one of the target activity types in this study, and log P for non‐ionized molecules, have often been employed as one of the physicochemical descriptors in traditional QSAR research. There are also many descriptors worth trying, such as quantum chemical descriptors and 3D descriptors [31, 32]. Unfortunately, the datasets used in this study were given as numerical information that has employed in competitions, so it is difficult to identify the molecules. If those descriptors could be replaced with the other descriptors, we could have quantitatively analyzed the efficiency of descriptors for the activity type.

Attempting the latest DNN techniques such as RNN with LSTM and CNN [5–8] may be possible to dramatically improve predictive performance. In particular, we focus on the graph convolutional neural networks. Neural Fingerprint [33], Gated Graph Sequence Neural Networks [34], WeaveNet [35], SchNet [36, 37] are introduced in the Chainer Chemistry library related to the ANN platform Chainer we adopted in this study. Now, we are applying them for our own QSPR study and will publish their results as soon as possible [38].

## 5 Conclusions

In order to develop an effective QSAR/DNN model using a general purpose ANN platform, we investigated DNN settings that could achieve the best predictive performance comparable to the champion teams by using the same datasets with the Kaggle QSAR competition. The Meister setting that showed overall better R2 performance for all 15 datasets was to use four hidden layers in the QSAR/DNN model, similar to the recommended setting of the champion team, but using smaller mini‐batch size was an important difference from the recommendation. The main reason seems that the Adam algorithm was adopted as the backpropagation optimization method implemented on the platform we used.

In our results, as with the champion team, the R2 performance of each target seems to be related to the active type. In fact, the log D activity type based on a comparison of two simple chemical processes showed the R2 predictive performance of over 0.8, even with a single hidden layer. The in vitro activity type based on a biological mechanism was roughly around 0.5 in R2 values, and almost targets of this type required at least four hidden layers. And bioavailability via multiple biological mechanisms showed the worst prediction accuracy. Thus, it can be said that the predictive performances of activity types except IC50 correlates qualitatively with the complexity of biological mechanisms or chemical processes in the molecular activity measurements. We presumed that the R2 performance of IC50 type may be dependent on the number of activity data measured under established experimental conditions, because IC50 is dependent on the concentration of the target enzyme and its substrate.

If the qualitative correlation between R2 performance and activity type is due to the complexity of biological mechanisms or chemical processes observed in activity measurements, it results in the limits of the molecular descriptors and the DNN models adopted here. If R2 performance has reached a limit where further efforts will not improve it, hence, we consider it to be at the limits of the molecular descriptors and the DNN models. In order to improve it, it may be possible to make DNN model more complicated by increasing the number of hidden layers and neurons, or to replace descriptors with higher‐order features. Unfortunately, in this study, such attempts could not be performed due to some circumstances mentioned above.

Now we are analyzing the results of further repeated optimizations with different random seeds for DNN initialization, and some inexplicit issues in this paper are becoming clear. In addition, we have prepared a large amount of molecular information and its activity data for other targets obtained from a public available database site, and are investigating some GCNN models. Details will be published elsewhere as soon as possible [38].

## Conflict of Interest

None declared.
